# Assessing Medical Students’ Perceptions of High-Fidelity Psychiatric Simulation

**DOI:** 10.1192/bjo.2025.10306

**Published:** 2025-06-20

**Authors:** Sita Shah, Gabrielle Sanders, Anita Stowell, Lucy Evans

**Affiliations:** 1 Cambridge and Peterborough NHS Foundation Trust, Cambridge, United Kingdom; 2 The Princess Alexandra Hospital, Harlow, United Kingdom

## Abstract

**Aims:** The use of high-fidelity simulation in psychiatry remains under-utilised. We aimed to evaluate the impact of high-fidelity psychiatric simulation on final-year medical students at two UK medical schools using a mixed-methods approach.

**Methods:** We delivered psychiatric simulation to final-year medical students using simulated patients, in a simulated medical ward or emergency department. Scenarios provided an integration between physical and mental health. Thirty-four students completed pre- and post-simulation questionnaires, rating their confidence in assessing patients with mental health problems, performing a suicide risk assessment, understanding different sections of the Mental Health Act and recognising bias towards patients with mental health problems on a 10-point Likert scale. Paired Likert data were analysed using Wilcoxon signed rank test, with correction for multiple comparisons with the false discovery rate (FDR). Ethical approval was sought from Queen Mary University of London to undertake a focus group, in which eleven students participated. Data were analysed using a reflexive thematic analysis technique using NVIVO 14.

**Results:** The simulation resulted in a statistically significant increase in students’ confidence in assessing patients with mental health problems (pFDR <0.001), performing suicide risk assessments (pFDR <0.001), recognising bias (pFDR <0.001) and understanding different Mental Health Act sections (pFDR <0.001). 100% of participants enjoyed the integration of physical and mental health and felt the scenarios were realistic. Over 90% wanted to see more psychiatric simulation in the undergraduate curriculum. The following themes were identified from the thematic analysis; 1) Student anxieties relating to psychiatry, 2) Current psychiatry teaching methods, 3) Recognising the utility of simulation and 4) Limitations of psychiatric simulation.

**Conclusion:** There is a gap in the undergraduate curriculum to incorporate high-fidelity psychiatric simulation. Final-year medical students found the simulation to be enjoyable and beneficial for learning. Future work should involve larger sample sizes, simulation of psychiatric emergencies and expansion into postgraduate teaching programmes. Subsequent curriculum evaluations should distinguish between high and low-fidelity simulation so we can accurately assess its implementation in UK medical schools.

